# Late gestation metabolizable energy intake is associated with modest differences in adipose tissue insulin responsiveness in antepartum beef cattle

**DOI:** 10.1093/jas/skaf421

**Published:** 2026-02-16

**Authors:** Koryn S Hare, Emily McKinlay, Katharine M Wood, Gregory B Penner, Michael A Steele

**Affiliations:** Department of Animal Biosciences, Animal Science and Nutrition, Ontario Agricultural College, University of Guelph, Guelph, ON N1G 1Y2, Canada; Department of Animal and Poultry Science, College of Agriculture and Bioresources, University of Saskatchewan, Saskatoon, SK S7N 5A8, Canada; Department of Animal Biosciences, Animal Science and Nutrition, Ontario Agricultural College, University of Guelph, Guelph, ON N1G 1Y2, Canada; Department of Animal Biosciences, Animal Science and Nutrition, Ontario Agricultural College, University of Guelph, Guelph, ON N1G 1Y2, Canada; Department of Animal and Poultry Science, College of Agriculture and Bioresources, University of Saskatchewan, Saskatoon, SK S7N 5A8, Canada; Department of Animal Biosciences, Animal Science and Nutrition, Ontario Agricultural College, University of Guelph, Guelph, ON N1G 1Y2, Canada

**Keywords:** adipose, beef cow, glucose-insulin kinetics, metabolizable energy

## Abstract

The objective of this study was to evaluate whether different metabolizable energy (**ME**) intakes would affect insulin responsiveness in late gestation beef cattle. Primiparous and multiparous cattle were fed rations that supplied 78% (**LowME**, *n* = 7 heifers and 12 cows) or 120% (**HighME**, *n* = 9 heifers and 10 cows) of predicted ME requirements from day −52 until calving, and then fed a common lactation ration. Body weight and rib and rump fat depth were measured every 2 wk prior to calving and on days 7, 13, 28, and 55 after calving. Plasma and serum were collected on day −3 and 7 relative to calving. Cattle underwent an intravenous glucose tolerance test (**IVGTT**) and a subcutaneous adipose biopsy on day −7 and −6, respectively. Prepartum body weight was similar (*P *= 0.62) for LowME and HighME cattle, but HighME cattle had greater (treatment by time: *P *= 0.01) rump fat depth than LowME at day −10 and −3 relative to calving. Overall, prepartum rib and rump fat depth tended to be greater (*P *= 0.07 and 0.06, respectively) for HighME vs. LowME. Glucose and insulin were similar (*P *≥ 0.19) during the IVGTT and serum NEFA were elevated (*P *< 0.01) for LowME. The HighME NEFA decrement was lesser (treatment-time: *P *= 0.03) than that of LowME, indicating reduced insulin responsiveness. Adipocyte area tended to be larger (*P *= 0.05) for HighME. Antepartum glucose and cholesterol were greater (*P *< 0.04) and serum NEFA was lower (*P *< 0.01) for HighME vs. LowME. Postpartum albumin, glucose, and cholesterol were all increased (*P *≤ 0.03) on day 7 after calving by feeding HighME before calving. Postpartum body weight was similar (*P *= 0.19) between treatments while rump fat depth was still less (*P *= 0.03) for LowME compared to HighME. The HighME cattle tended to have more (*P *= 0.06) rib fat depth postpartum. As such, HighME provision during late gestation improved markers of energy balance and was associated with modest reductions in antepartum insulin responsiveness, but this had few impacts after calving.

## Teaser

Evidence is presented which suggests that the degree to which late pregnant beef cattle respond to insulin is tied to how well their rations meet their energy requirements before calving. This information can be used to refine nutritional models for late gestation beef cattle.

## Introduction

Late gestation glucose requirements increase substantially to support fetal growth and colostrogenesis (Bauman and Currie, 1980) and may result in a glucose deficit immediately prior to calving (Overton, 1998; Douglas et al., 2006). In ruminants, glucose is predominantly supplied by hepatic gluconeogenesis using propionate as a precursor with intestinal glucose absorption only marginally contributing to the glucose pool (Huntington et al., 2006; Aschenbach et al., 2010). Diets that provide fewer readily fermentable carbohydrates, such as those provided to late gestation beef cattle (NASEM, 2016; Kerwin et al., 2022), may increase the severity of a glucose deficiency prior to calving by limiting substrate availability for hepatic gluconeogenesis. Further, starch digestibility is reduced as beef heifers approach calving (Hare et al., 2019b), imposing an additional challenge in maintaining gluconeogenic substrate supply. Thus, while it is probable that beef cattle experience an antepartum glucose deficit, they may be employing alternative metabolic adaptations either pre- or postpartum to mitigate putative deficits in glucose supply.

Female mammals prioritize accessory biological functions, such as pregnancy and lactation, above their own maintenance requirements by repartitioning substrate utilization through hormonal regulation (Bauman and Currie, 1980). Reduced insulin responsiveness (**IR**) is one of the endocrine adaptations that facilitates the partitioning of glucose to the conceptus (Bauman and Currie, 1980; Baumgard et al., 2017), but IR can be modulated by physiological and nutritional factors before calving and it can be exacerbated to the point that homeorhesis becomes maladaptive. Both excessive adiposity (Jaakson et al., 2018; Zhang et al., 2019; Angeli et al., 2021) and global nutrient overconsumption (Schoenberg and Overton, 2011; Schoenberg et al., 2012; Salin et al., 2017) can exacerbate prepartum adipose-specific insulin resistance in dairy cattle. But, in beef cattle, there is limited comparable work evaluating IR using intravenous glucose tolerance tests (**IVGTT**). Overfeeding metabolizable protein (**MP**) to late gestation beef cattle altered some IVGTT parameters but did not influence glucose and insulin concentrations during the test (Hare et al., 2022), suggesting that prepartum nutrient supply may also influence insulin dynamics in beef cattle. However, to our knowledge, previous work has not evaluated how metabolizable energy (**ME**) intake prior to calving affect IR in beef cattle.

We hypothesized that greater ME intake prior to calving would reduce antepartum IR of beef cattle, compromising postpartum performance. Our objective was to evaluate how different ME intakes affect glucose-insulin kinetics and NEFA concentrations during an antepartum IVGTT and peripartal inflammatory and hepatic markers of metabolic dysregulation.

## Materials and Methods

The experiment was conducted from January to November 2021 at the Ontario Beef Research Center (University of Guelph, Guelph, ON) in accordance with Canadian Council of Animal Care guidelines and approved by the University of Guelph (Guelph, ON) Animal Care Council (Animal Utilization Protocol #4419).

### Experimental design, cow husbandry, and dietary treatments

Simmental-Angus crossbred cattle (*n* = 16 heifers and 22 cows) from the Ontario Beef Research Center (University of Guelph, Guelph, ON) were bred by fixed-time artificial insemination (non-sexed commercially available Angus semen, 9 different sires of similar genetic merit). For 2 wk in advance of the study, cattle were acclimated to a control diet supplying 104% ME and 121% of predicted MP requirement (detailed in Kladt et al., 2025) before being assigned at random to be fed rations from day −52_** ± **_5.1 (mean _**±**_ SD) relative to calving that under- (*n* = 7 heifers and 12 cows, 78% ME; **LowME**) or oversupplied (*n* = 9 heifers and 10 cows, 120% ME; **HighME**) ME respective to predicted requirements (CNCPS 6.55; Nutritional Dynamic Software, RUM&N Sas, Reggio Emilia, Italy). Metabolizable protein was supplied at or above predicted requirements for LowME (97% MP) and HighME (124% MP), respectively. The necessary replication was determined *ad hoc* using PROC POWER of SAS 9.4 (SAS Institute, Cary, NC) using data from Hare et al. (2022) as a reference for mean and variation in glucose and insulin concentrations throughout the IVGTT. It was determined that 20 replicates per treatment would provide adequate statistical power (1 – ß = 80%; _**ɑ**_ = 0.05) to detect a 10% difference in overall plasma glucose concentrations and 30% effect size in plasma insulin concentrations. One LowME heifer and one HighME cow were removed from the experiment due to complications at calving.

Cattle were housed in pens (5.9_** × **_13.4 m^2^; *n* = 4 or 5 heifers/pen, *n* = 6 cows/pen) bedded with straw. The 19 LowME cattle were sourced from 9 pens (4 pens with heifers and 5 pens with cows), whereas the 19 HighME cattle were sourced from 8 pens (3 pens with heifers and 5 pens with cows). All cattle within a pen received the same treatment provided in three Insentec feed bunks (Hokofarm Group, Marknesse, the Netherlands). Treatments were formulated with CNCPS 6.55 (Nutritional Dynamic System software, RUM&N Sas, Reggio Emilia, Italy) to supply 80 or 120% of predicted ME requirements of cattle at 270 d in gestation that were losing 2 BCS points (on a scale of 1 to 9) over 100 d prior to calving. The Ontario Beef Research Center herd averages (breed: Angus; second parity; BW: 715 kg; and a BCS of 7 on a scale of 1 to 9) and an anticipated calf birth BW of 38 kg were specified within the model to predict ME requirements. The herd averages were input into the model as the rations were formulated to feed the entire herd, of which a uniform subset of younger cattle were selected to perform IVGTT immediately prior to calving. For comparison, the IVGTT subset was first (45%), second (26%), and third (29%) parity cattle with heifers and cows having respective initial BW of 633_** ± **_10.4 kg and 719_** ± **_18.3 kg (mean _**±**_ SE). At 270 d gestation, the IVGTT subset heifers and cows weighed 640_** ±**_9.4 and 740.7_** ± **_18.2 kg, respectively. Initial BCS was 6.8_** ± **_0.14 for heifers and 7.2_** ± **_0.11 for cows and, at day 270, BCS was 6.5_** ± **_0.12 for heifers and 6.4_** ± **_0.44 for cows. Calf birth BW were 31.9_** ± **_1.08 and 36.7_** ± **_0.78 kg (uncorrected raw means) for heifers and cows, respectively. Treatments were fed to allow for DMI equating to 1.35% and 1.45% of BW for LowME and HighME treatments, respectively, to ensure that cattle consumed their targeted ME provision of 80% or 120% of predicted requirements. An inherent consequence of this design is that the differences in DM and ME intake altered the predicted MP supply for LowME and HighME treatments. To account for any potential impact of dietary ME intake on protein supply, we opted to formulate diets to isonitrogenous (10.6% to 10.7% DM as CP) by including urea in the LowME ration. Feed was supplied once per day between 0900 and 1100 h. Ingredient and chemical composition of the diets are shown in Table 1. Insentec bunks were programmed to prevent cattle from consuming more feed than provided relative to their BW. Once an individual cow has consumed her daily feed allocation, the Insentec bunk’s Intelligent Feeding Gate closed and prevented her re-entry into the feed bunk until the following day. Daily feed provision was programmed manually through the software and adjusted every 2 wk corresponding to BW measurement (described below) and the running average in ingredient DM to ensure that DM provision relative to BW remained consistent throughout the feeding period. Based on observed DMI and chemical analysis from feed samples collected throughout the experiment (described below), LowME and HighME cattle consumed 78% and 120%, respectively, of their predicted ME requirements (retrospectively modeled using CNCPS 6.55; Nutritional Dynamic System software, RUM&N Sas, Reggio Emilia, Italy). Supplementary Tables S1 and S2 provide retrospective CNCPS model-predicted ME and MP requirements, supplies, and sufficiency of ME and MP supply relative to requirements from for 56 d prior to calving.

**Table 1.** Ingredient composition, chemical composition, and the metabolizable energy (ME) and protein (MP) concentrations of rations formulated to provide 80 (LowME, *n* = 19), or 120% (HighME, *n* = 19) of predicted ME requirements for 52 d prior to calving^1^

	Prepartum treatment	
Parameter	LowME	HighME
**Ingredient, %DM**		
**Haylage**	5.0	5.0
**Corn silage**	5.0	5.0
**Chopped dry hay**	30.5	44.5
**Chopped wheat straw**	55.0	5.0
**High moisture corn grain**	–	18.5
**Whole corn grain**	–	18.5
**Mineral-vitamin premix^2^**	3.5	3.5
**Urea**	1.0	–
**Dry matter, %**	84.5	80.8
**Chemical composition, %DM**		
**CP**	10.6 ± 0.23	10.7 ± 0.17
**RDP**	7.3	6.5
**aNDFom^3^**	67.4 ± 0.48	36.8 ± 0.41
**Starch**	3.0 ± 0.03	27.1 ± 0.17
**Ether extract**	2.2 ± 0.04	3.0 ± 0.06
**Ca**	0.7 ± 0.01	0.7 ± 0.01
**P**	0.3 ± 0.00	0.4 ± 0.01
**Mg**	0.2 ± 0.00	0.3 ± 0.00
**K**	1.3 ± 0.02	1.6 ± 0.03
**ME, Mcal/kg**	1.56	2.59
**ME Supply^4^, % requirement**	78.4	120.3

1 All cattle received the same ration after calving.

2 The mineral-vitamin premix was formulated by Floradale Feed Mill Limited (Floradale, ON); chemical composition: 293.4 kIU/kg vit A, 67.1 kIU/kg vit D, 1,364.6 kIU/kg vit E, 447.0 ppm Cu, 1,586.4 ppm Fe, and 3,866.6 ppm Zn.

3 Amylase- and sodium sulfite-treated NDF corrected for ash content.

4 Rations were formulated and ME and MP requirements were predicted using CNCPS 6.55 (Nutritional Dynamic System software, RUM&N Sas, Via Sant’ Ambrogio, Italy) and reassessed post-experiment to determine accuracy respective to initial ME and MP formulations.

In the 2 wk preceding the anticipated date of calving (282-d post artificial insemination), all cattle were carefully monitored 24 h/d by either research personnel or Ontario Beef Research Center technicians for signs of parturition. The time of calving was recorded immediately after the calf was born and the cow-calf pair were given up to an hour to bond prior to measurements, including colostrum collection, and blood sampling (reported elsewhere). Cow-calf pair were housed in maternity pens (3.4_** × **_13.4 m^2^) for 5 to 7 d after calving prior to being group-housed [pens: 17.7_** × **_13.4 m^2^; *n* = 18 cow-calf pairs (mixed treatments) per pen] and, whether individually- or group-housed, were fed a common lactation diet for *ad libitum* intake in Insentec feed bunks (9 feed bunks per pen). The postpartum ration was imposed by the Ontario Beef Research Center and was privately formulated (Floradale Feed Mill Ltd, Floradale, ON). It was composed of haylage (36.2%DM), corn silage (23.6%DM), wheat straw (35.2%DM), and a mineral vitamin premix (5%DM). The chemical composition (DM basis) was 9.3% crude protein, 53.8% amylase- and sodium-sulfite treated neutral detergent fiber, 9.1% starch, 2.8% ether extract, 0.6% calcium, and 0.2% phosphorous, and it supplied 2.09 Mcal ME/kg DM (approximately 105% predicted ME requirements) and 1,170 g MP/d.

### Data curation

#### Dry matter intake and feed sampling

Daily as-fed feed intake per cow was recorded by the Insentec feed bunks (Roughage Intake Control system; Hokofarm Group, Marknesse, the Netherlands), corrected for DM and chemical composition, summarized by week relative to calving, and the overall pre- and postpartum period DMI was averaged by pen. Fermented forages (haylage and corn silage) were sampled (1 to 2 kg) twice weekly, dry forages (wheat straw and chopped dry hay) were sampled (1 kg) weekly, and concentrates (high-moisture corn grain, whole corn grain, vitamin-mineral premix, and urea) were sampled (1 kg) every 2 wk to measure DM. Dried feed samples were ground through a 1-mm screen using a hammer mill and either composited by week (fermented forages) or analyzed as is for chemical composition (A&L Laboratories Inc., London, ON).

#### Body weight and rib and rump fat thickness

Cattle were weighed at the start of the experimental period (day −52_** ± **_5.1 relative to calving [mean _**±**_ SD]) and every 2 wk thereafter (day −38_** ± **_4.2, −24_** ± **_4.0, −10_** ± **_2.4, and −3.1_** ± **_2.1). Body weight was recorded prior to feeding between 0800 to 1100 h. Rib and rump fat depth were measured by ultrasonography (ExaGo; 18 cm 3.5 Mhz linear transducer; Echo Control Medical, Angoul_**ê**_me, France; Broring et al., 2003) on days corresponding to the first 4 BW measurements. The ultrasound probe was placed between the 12^th^ and 13^th^ rib to measure rib fat depth. After calving, BW was recorded on days 7_** ± **_1.7, 13_** ± **_2.0, 26_** ± **_2.6, and 54_** ± **_2.8, and rib and rump fat depth ultrasonographic measurements were performed on the latter three timepoints.

#### Plasma and serum collection and analyses

Coinciding with weigh-days, venous blood was drawn via jugular venipuncture at day −3 and 7 relative to calving (day 0) to characterize the antepartum and postpartum metabolic profiles, respectively. Blood used to harvest plasma was collected into a vacutainer that contained an anticoagulant (158 IU of heparin; BD B366480, Becton Dickinson, Franklin Lakes, NJ), and a competitive serine protease inhibitor (5_** µ**_g aprotinin/L; Sigma-A1153, Millipore-Sigma, Oakville, ON) was immediately added to blood. Then, plasma was separated by centrifugation (3,000_** × **_*g* at 4_** °**_C for 15 min). Blood used for serum isolation was collected into a vacutainer that did not contain anticoagulant (BD B366430, Becton Dickinson, Franklin Lakes, NJ) and was allowed to clot at room temperature for 30 min prior to centrifugation (3,000_** × **_*g* at 4_** °**_C for 15 min). Three 1.5-mL aliquots of plasma and serum were immediately frozen at −20 _**°**_C until analysis.

Serum was photometrically analyzed for total protein, albumin, globulin, urea, beta-hydroxybutyrate (**BHBA**), non-esterified fatty acids (**NEFA**), haptoglobin, glucose, cholesterol, aspartate aminotransferase (**AST**), gamma-glutamyl transferase (**GGT**), and glutamate dehydrogenase (**GLDH**) by the Animal Health Laboratory (University of Guelph, Guelph, ON) using a Cobas 6000 series analyzer (Roche Diagnostics International Ltd, Rotkreuz, Switzerland). Plasma insulin was analyzed in duplicate with a commercially available ELISA (Alpco Bovine Insulin ELISA, Salem, NH, USA) with inter- and intra-assay CV of 12.1% and 3.2%, respectively.

#### Intravenous glucose tolerance test

Intravenous glucose tolerance tests were performed on day −7_** ± **_3.5 (mean _**±**_ SD) relative to calving. Feed was withheld for at least 18 h prior to the start of the test (1400 to 1500 h) by programming the Insentec feed bunks to prevent cattle from accessing feed. Cattle were weighed and then restrained in a chute and fit with an 8.3 cm 16-gauge indwelling jugular catheter (BD Angiocath IV Catheter #382258, Becton Dickinson, Mississauga, ON, Canada). A 50% dextrose solution (2.73 *M* glucose; Dextrose 50%, V_**é**_toquinol, Cambridge, ON, Canada) was used to dose glucose (1.36 g glucose/kg BW^0.75^; Joy et al., 2017; Hare et al., 2022). The glucose was infused over 60 seconds at the start of the IVGTT and the catheter line was immediately flushed with 20 mL of heparinized saline (100 IU heparin/mL in 0.9% NaCl; Baxter JB1324, Baxter International, Mississauga, ON). Blood (10 mL) was drawn and discarded and then blood was sampled. Following sampling, the catheter was flushed with 10 mL heparinized saline. Blood (10 mL) was collected at −3, 0 (immediately after infusion), 3, 6, 9, 12, 15, 20, 25, 30, 45, 60, and 90 min relative to the glucose bolus. The duration of sampling (90 min) was chosen to minimize the timeframe during which cattle were restrained, since Hare et al. (2022) demonstrated that gestating beef heifer plasma glucose and insulin concentrations equilibrate to baseline concentrations by 90 min post-glucose bolus. Blood was immediately transferred to vacutainers that contained either 158 IU of heparin (plasma; BD B366480, Becton Dickinson, Franklin Lakes, NJ) or no anticoagulant (serum; BD B366430, Becton Dickinson, Franklin Lakes, NJ). Blood intended for plasma collection immediately had a competitive serine protease inhibitor added, as previously described. Plasma vacutainers were placed on ice while serum vacutainers were allowed to clot for 30 min at room temperature prior to centrifugation (3,000_** × **_g at 4_** °**_C for 15 min) to separate the supernatant. Plasma and serum were placed in three 1.5-mL aliquots frozen at −20 _**°**_C until analysis.

Plasma glucose concentration was analyzed using the glucose oxidase/peroxidase reaction and colorimetric detection (inter-assay CV: 4.95%; intra-assay CV: 2.06%; Trinder, 1969) and insulin concentrations were analyzed with a commercially available ELISA (inter-assay CV: 4.33%; intra-assay CV: 3.51%; Alpco Bovine Insulin ELISA, Salem, NH, USA) according to the manufacturer’s directions. Serum NEFA concentrations were colorimetrically detected using the NEFA-HR(2) kit (FUJIFILM Wako Chemicals, Richmond, VA). Inter- and intra-assay CV for serum NEFA were 1.93 and 1.04%, respectively. All samples were measured in duplicate and accepted when the CV was <5.0 (glucose and NEFA) or 7.0% (insulin).

#### Adipose biopsies

On the day following the IVGTT (day 6_** ± **_3.5 relative to calving), the cattle were restrained in a chute to biopsy subcutaneous adipose tissue at 1200 h. The biopsy site (caudal portion of the tail-head) was shaved and sanitized with 4% chlorohexidine and 70% ethanol alcohol. All biopsy supplies (scalpel handle, forceps, scissors, and gauze) were sterilized by autoclaving. Lidocaine HCl 2% (6 mL) was used to anesthetize the biopsy site, and a 3-cm incision was created using a scalpel. Subcutaneous adipose tissue (approximately 5 g) was excised with forceps and scissors (Añez-Osuna et al., 2019) and rinsed with sterilized phosphate-buffered saline. Adipose tissue was transferred to two 1.5-mL cryovials (_**∼**_500 mg/vial), immediately snap-frozen in liquid N_2_, and then transferred to a −80 _**°**_C freezer for storage until analysis. The remaining tissue was fixed in 40 mL of 10%-buffered formalin for 48 h and then stored in 70% ethanol alcohol for histological analysis.

**Figure 1.**
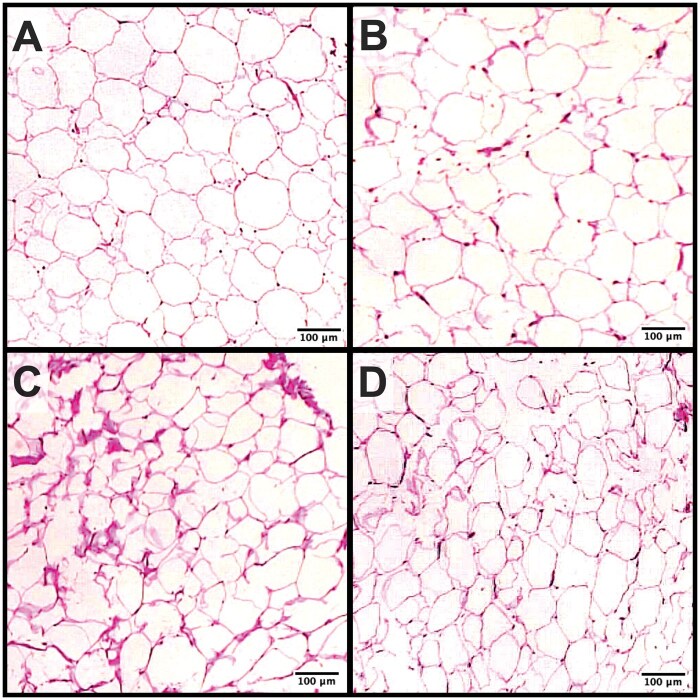
Brightfield microscopy images (4× original magnification) of tailhead adipose tissue from cattle that had consumed rations formulated to supply either 80% (**LowME**) or 120% (**HighME**) of predicted metabolizable energy requirements prior to calving. Adipose biopsies were performed 6 d prior to calving. Panels are as follows: (A) HighME, multiparous cattle; (B) HighME, primiparous cattle; (C) LowME, multiparous cattle; and (D) LowME, primiparous cattle. Adipose tissue is stained with hematoxylin and eosin for contrast.

Fixed adipose tissue was sectioned (5_** µ**_m) onto glass slides and stained with hematoxylin and eosin (Animal Health Laboratory, University of Guelph, Guelph, ON). Slides were imaged at 4_**×**_ magnification (Leica ICC50W; Leica Microsystems, Wetzlar, Germany) and at least 4 images were captured of different section areas. The area and diameter of 100 adipocytes were measured (ImageJ 1.46r; National Institutes of Health, Bethesda, MD) from randomly selected fields between images and averaged, as previously described (Akter et al., 2011; Locher et al., 2015). Representative images of adipocytes from HighME and LowME heifers and cows are presented in Figure 1.

### Calculations and statistical analyses

Prepartum BW was corrected for the weight of the conceptus (conceptus-corrected BW [**CCBW**]) as estimated by the equation detailed in NASEM (2016): CW = CBW _**×**_ 0.01828 _**× ℮**_^(0.02 × DP–0.0000143 × (DP × DP))^, where CW is the conceptus weight (kg), CBW is the calf birth weight (kg) and DP is days pregnant.

From the blood analyte concentrations during the IVGTT, several variables were calculated to describe the kinetics of the plasma glucose, insulin, and serum NEFA curves: Baseline (−3 min relative to glucose bolus), maximum (**C_max_**), and the difference between C_max_ and baseline (**ΔC_max_**) glucose and insulin concentrations and the time at which C_max_ occurred (**T_max_**) were determined. For serum NEFA, baseline and minimum (**C_min_**) NEFA concentrations, and the time to C_min_ (**T_min_**) were determined. Glucose clearance rate was calculated as the slope of the natural logarithm of glucose from 3 to 45 min (%/min; Kaneko, 1997) using the slope function in Microsoft Excel (Version 16.69.1; Microsoft Excel for Mac, Microsoft Corporation, Redmond, WA). As described in Pacini et al. (2009), this interval represents the second phase (main elimination interval) in a 3-phase exponential decay model of glucose concentrations over time. Insulin clearance rate was calculated as the slope of the natural logarithm of insulin from T_max_ until 90 min using the slope function of Microsoft Excel (Version 16.69.1; Microsoft Excel for Mac, Microsoft Corporation, Redmond, WA). Non-esterified fatty acid disappearance rate (mEq/L _**×**_ min^−1^) was calculated as the slope (Microsoft Excel version 16.69.1; Microsoft Excel for Mac, Microsoft Corporation, Redmond, WA) from −3 to 90 min relative to the glucose bolus. Total glucose and insulin area-under-the-curve from 0 to 90 min (**AUC_0to90min_**) were calculated by the trapezoidal sum of measurement intervals (Chiou, 1978). The positive incremental area-under-the-curve from 0 to 90 min (**I-AUC_0to90min_**; Cardoso et al., 2011) that accounts for baseline analyte concentrations was also calculated for glucose and insulin concentrations. The NEFA decremental AUC from 0 to 90 min (**dAUC_0to90min_**) was calculated using the trapezoidal sum of NEFA concentrations during the time interval. Decremental NEFA concentration throughout the IVGTT was corrected by the baseline NEFA concentration. The glucose-stimulated reduction in serum NEFA (**GSRN**) was determined as the percentage reduction in serum NEFA concentrations from the baseline, as detailed in Schoenberg et al. (2012). The insulin sensitivity index (insulin’s ability to increase glucose disappearance; Bergman et al., 1979, 1987) was calculated as shown in Hare et al. (2022) using the formula by Galvin et al. (1992) as modified by Pacini et al. (2009).

The residual distributions of all data were assessed using PROC Univariate (SAS 9.4; SAS Institute Inc., Cary, NC) to confirm or reject that data were normally distributed (Shapiro-Wilk *P *> 0.05). When non-Gaussian residual distributions (Shapiro-Wilk *P *_**≤**_ 0.05) were detected, data were transformed using either an inverse (antepartum GLDH, antepartum insulin, postpartum haptoglobin, total glucose AUC, and insulin sensitivity index) or a logarithmic (prepartum BW and conceptus-corrected BW, postpartum BW, postpartum GLDH) transformation. Means from these variables are presented as the back-transformed mean and the 95% CI rather than the SE. Residual variance between treatments was tested using the COVTEST statement in PROC GLIMMIX and confirmed homoscedastic when residual homogeneity *P* > *x*^2^ was < 0.05. Heteroscedasticity was controlled using the GROUP= command in the RANDOM statement of PROC GLIMMIX to specify that residual variance be grouped by treatment within the model.

Once residual distribution had been assessed and adjusted for, the data were analyzed as a completely randomized design with PROC GLIMMIX. Non-repeated measurements were analyzed with the following model:


$${\text{y}}_{ijkl}=\mu +{\text{trt}}_{i}+{\text{parity}}_{j}+{\left(\text{trt}\times \text{parity}\right)}_{ij}+{\text{pen}}_{k}+\varepsilon_{ijkl}$$

where y_*ijkl*_ is the *l*^th^ observation of the *k*^th^ pen nested with the *i*^th^ treatment of the *j*^th^ parity, *µ* is the overall mean, trt_*i*_ is the fixed effect ot the *i*^th^ treatment, parity_*j*_ is the fixed effect of the *j*^th^ parity, pen_*k*_ is the random effect of the *k*^th^ pen, and ɛ_*ijkl*_ is the associated random error. Measurements repeated over time additionally included the fixed effects of the *m*^th^ time (minute or day) and interactions of the *i*^th^ treatment, *j*^th^ parity, and *m*^th^ minute according to:


$$y_{\mathrm{ijklm}}=µ+{\;\text{trt}}_{i}+{\;\text{parity}}_{j}+{\;\text{time}}_{m}+{\left(\text{trt}\times \text{parity}\right)}_{ij}+{\left(\text{trt}\times \text{time}\right)}_{im}+{\left(\text{parity}\times \text{time}\right)}_{jm}+{\left(\text{trt}\times \text{parity}\times \text{time}\right)}_{\mathrm{ijm}}+{\;\text{pen}}_{k}+\varepsilon_{\mathrm{ijklm}}$$

Covariance-time plots were generated in using PROC GPLOT to visually assess variance similarity, the presence of decay over time, and correlation over time (Stroup, 2016), as well as guide the selection of the appropriate covariance structure. For measurements repeated over time, covariance was modeled with heterogenous compound symmetry, compound symmetry unstructured, and ante-dependence (1) covariance structures and best-fit was determined independently for each variable based on the lowest AIC and BIC without the indication of over- or under-dispersion. Degrees of freedom were approximated within the model using Kenward-Rogers to account for unbalanced observations between treatments. Non-repeated and evenly spaced measures were adjusted with Tukey’s, whereas Games-Howell’s multiple comparison test was specified for unevenly spaced repeated measurements (Lee and Lee, 2018). Differences were declared when *P *< 0.05 and tendencies were considered when 0.05 ≤ *P *< 0.10. Data are expressed as mean ± SE.

## Results

### Pre- and postpartum DMI, body weight, and adiposity

Cattle receiving HighME consumed more (*P *≤ 0.03; Table 2) DM and had greater DMI represented as a percent of BW prepartum than those fed LowME. Heifers had lower (*P *= 0.02) DMI than cows, but their DMI as a percentage of BW did not differ (*P *= 0.60). The interaction between treatment and parity did not affect (*P *≥ 0.10) prepartum DMI or DMI represented as a percent of BW. Calf birth BW did not differ (*P *= 0.19) between treatments. After calving, DMI and DMI as a percentage of BW were not affected (*P *≥ 0.27) by prepartum treatment or the treatment × parity interaction. While heifers still consumed less (*P *< 0.01) DM than cows, their DMI represented as a percentage of BW was not different (*P *= 0.25).

**Table 2.** Mean DMI, BW and conceptus-corrected BW (CCBW), adiposity metrics, and calf birth BW of cattle fed rations formulated to be deficient (LowME = 80% ME requirement, *n* = 19) or in excess (HighME = 120% ME requirement, *n* = 19) of predicted metabolizable energy requirement during late gestation for 52 d^1^^,^^2^

	Treatment			Parity			*P*-value^3–5^					
Parameter^6^	LowME	HighME	SE	Heifers	Cows	SE	TRT	Parity	Day	T × P	T × D	P × D
**Prepartum**												
**DMI, kg**	8.8	10.0	0.37	8.8	10.1	0.37	0.03	0.02	–	0.78	–	–
**DMI, %BW**	1.30	1.45	0.014	1.37	1.37	0.014	<0.01	0.60	–	0.10	–	–
**BW^7^, kg**	679	695	(638–741)	640	737	(596–779)	0.62	0.01	<0.01	0.58	<0.01	0.01
**CCBW^7^, kg**	643	654	(602–699)	604	696	(561–737)	0.72	0.01	<0.01	0.58	<0.01	0.01
**Rib fat depth, mm**	6.0	8.5	0.93	6.8	7.7	0.97	0.07	0.48	<0.01	0.08	0.62	<0.01
**Rump fat depth, mm**	10.6	15.1	1.64	11.1	14.6	1.72	0.06	0.14	0.01	0.08	0.01	0.05
**Adipocyte area, µm^2^**	6,345.5	7,870.8	504.6	6,691.5	7,524.8	504.6	0.05	0.25	–	0.80	–	–
**Adipocyte diameter, µm**	85.0	92.9	6.0	83.4	94.4	6.0	0.36	0.21	–	0.45	–	–
**Calf birth BW, kg**	33.5	35.9	1.25	31.7	37.7	1.31	0.19	<0.01	–	0.46	–	–
**Postpartum**												
**DMI, kg**	15.6	15.0	0.35	13.7	16.8	0.36	0.27	<0.01	–	0.65	–	–
**DMI, %BW**	2.30	2.23	0.100	2.18	2.35	0.105	0.61	0.25	–	0.62	–	–
**BW, kg**	658	703	23.8	632	728	24.9	0.19	0.01	0.47	0.35	0.41	0.58
**Rib fat depth, µm**	4.6	7.1	0.95	5.2	6.4	0.99	0.08	0.35	0.83	0.05	0.78	0.42
**Rump fat depth, µm**	7.6	13.8	2.13	8.6	12.8	1.86	0.03	0.12	0.52	0.06	0.46	0.99

1 All cattle received the same ration after calving.

2 CCBW (NASEM, 2016).

3 Treatment (TRT), the treatment by parity interaction (T × P), the treatment by day interaction (T × D), and the parity by day interaction (P × D)

**Figure 2.**
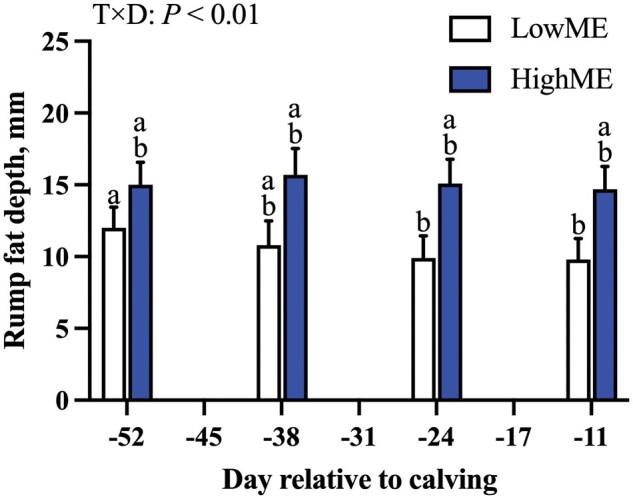
The effect of the treatment by day (**T × D**, *P *< 0.01) on prepartum rump fat depth. Cattle were fed rations that were formulated to supply 80% (**LowME**) or 120% (**HighME**) of predicted metabolizable energy requirements for 52 d prior to calving. Data are presented as means ± SE. ^a,b^Means that do not share a common superscript are different (*P *< 0.05).

4 Interactions are provided in Figure 2 (T × D interaction, prepartum rump fat depth), Supplementary Figure S1 (T × D and P × D interactions, prepartum BW and CCBW; see online supplementary material for a color version of this figure), and Supplementary Figure S2 (P × D interaction, prepartum rib fat depth; see online supplementary material for a color version of this figure).

5 The 3-way interaction between treatment, parity, and day was included in the model but was not significant (*P *≥ 0.14) and *P*-values are not shown.

6 Dry matter intake was measured weekly from −52 to 0 (prepartum) and 0 to 52 d (postpartum) relative to calving. Cattle were weighed on day −52, −38, −24, −10, and −3 (prepartum) and day 7, 13, 26, and 54 (postpartum) relative to calving. Rib and rump fat depth was measured on day −52, −38, −24, and −11 (prepartum) and day 11, 26, and 54 (postpartum) relative to calving. Adipose biopsies were performed on day −6 relative to calving.

7 Data were normalized using a logarithmic transformation. The presented means are back-transformed. The 95% CI in shown in place of the SE.

Although prepartum BW and conceptus-corrected BW were not different (*P *≥ 0.34) between LowME and HighME cattle or due to the treatment × parity interaction, BW for HighME cows increased (treatment × day: *P *< 0.01, sliced by treatment: *P *≤ 0.01; Supplementary Figure S1A, see online supplementary material for a color version of this figure) as calving approached, whereas BW for LowME cows did not change prior to calving. When corrected for the weight of the conceptus, LowME cattle were lighter (treatment × day: *P *< 0.01, sliced by treatment: *P *< 0.01; Supplementary Figure S1C, see online supplementary material for a color version of this figure) on day −3 and −10 relative to day −24 and −38 while HighME cattle increased BW on day −3, −10, and −24 respective to day −38. Prepartum ME intake did not affect (treatment × parity: *P *≥ 0.58) BW or conceptus-corrected BW differently between cows and heifers, but cows weighed 21% more (*P *= 0.01) than heifers and had 20% greater (*P *= 0.01) conceptus-corrected BW. Heifers weighed their least (parity × day: *P *= 0.01, sliced by parity: *P *< 0.01; Supplementary Figure S1B, see online supplementary material for a color version of this figure) on day −38 and were heaviest on day −10 and −3, whereas cows were at their lowest BW on day −52 and increased in weight until day −24, at which point their weight did not change. Conceptus-corrected BW for heifers was lowest (parity × day: *P *= 0.01, sliced by parity: *P *< 0.01; Supplementary Figure S1D, see online supplementary material for a color version of this figure) on day −38 relative to day −52 and −24. By day −10 and −3, heifer conceptus-corrected BW was similar to day −52, −38, and −24. Cow CCBW was lower on day −52, −38, and −10 compared to day −24, and their CCBW on day −3 was similar to day −52, −38, −24, and −10. After calving, cow BW was 14% greater (*P *= 0.01) than heifers. Postpartum BW was not different (*P *≥ 0.19) by treatment on the treatment × parity interaction and did not change (day: *P *= 0.47) throughout the postpartum period or differ (interaction: *P *≥ 0.41) by the interaction of treatment or parity with day. The three-way interaction of treatment, parity, and day did not affect (*P *≥ 0.18; *P*-values not shown) prepartum BW, conceptus-corrected BW, or postpartum BW.

The LowME cattle lost (treatment × day: *P *= 0.01; Figure 2) rump fat depth on day −24 and −11 relative to −52, resulting in a loss (sliced by treatment: *P *< 0.01) of 2.2 ± 1.65 mm of rump fat depth from day −52 to −11. Because of this loss, LowME rump fat depth was less (sliced by day: *P *≤ 0.04) than HighME on day −24 and −11 by 5.6 and 5.2 ± 1.72 mm, respectively. Cattle fed HighME did not lose (sliced by day: *P *= 0.41) rump fat depth prior to calving. Overall, HighME cattle tended to have greater rib fat depth (*P *= 0.07) and rump fat depth (*P *= 0.06) than LowME cattle prior to calving. The loss of adipose reserves at the rump was reflected by adipocyte area tending to be less (*P *= 0.05; 19% reduction in adipocyte area) for LowME than HighME cattle on day −6 relative to calving. Adipocyte area was similar (*P *≥ 0.25) between parities and by the interaction of treatment and parity, and adipocyte diameter did not differ (*P *≥ 0.21) by the effect of treatment, parity, or the treatment by parity interaction. The interaction of treatment and day did not affect (*P *= 0.62) prepartum rib fat depth.

Prepartum rib fat depth was affected (interaction: *P *= 0.01; Supplementary Figure S2, see online supplementary material for a color version of this figure) by the parity × day interaction as it decreased for heifers as they approached calving, but it remained similar across days for cows during the prepartum period. The treatment by parity interaction tended to affect (*P *= 0.08) both prepartum rib and rump fat depth, but overall, they did not differ (*P *≥ 0.14) between parities. The three-way interaction of treatment × parity × day did not affect (*P *≥ 0.34) prepartum rib or rump fat depth.

In the postpartum period, HighME rump fat depth was 1.82× greater (treatment: *P *= 0.03) on average than that of LowME, and postpartum rib fat depth tended to be increased (*P *= 0.08) for HighME relative to LowME. The treatment by parity interaction tended to affect both rib (*P *= 0.05) and rump fat depth (*P *= 0.06). Heifers and cows did not differ (*P *≥ 0.12) in their rib and rump fat depth after calving. Further, the two-way interactions of treatment × day and parity × day did not affect (*P *≥ 0.42) postpartum rib and rump fat depth, and nor were these variables affected (*P *≥ 0.14) by the three-way interaction of treatment × parity × day.

### Ante- and postpartum blood analytes

Serum cholesterol and glucose concentrations were both elevated (*P *≤ 0.04; Table 3) 3 d prior to calving for HighME relative to LowME. In contrast, LowME cattle had serum urea and NEFA that were elevated (*P *< 0.01) compared to HighME. Serum albumin, GTT, AST, GLDH, BHBA, and haptoglobin and plasma insulin concentrations did not differ (*P *≥ 0.23) between treatments. Compared to cows, heifers had elevated (*P *= 0.02; 8.8% increase) serum glucose and tended to have greater (*P *= 0.08; 69% increase) serum GLDH concentration. Serum BHBA was lower (*P *= 0.01; 24.7% reduction) and serum NEFA tended to be lower (*P *= 0.07; 23.2% reduction) for heifers compared to cows. Tendencies for treatment × parity interactions were identified for serum glucose (*P *= 0.09) and urea (*P *= 0.07) concentrations, but no differences (*P *≥ 0.10) due to the interaction of treatment and parity were detected for the remaining blood analytes.

**Table 3.** Blood analytes at day −3 and 7 relative to calving from cattle that were fed rations for that were formulated to be deficient (LowME = 80% requirement, *n* = 19) or in excess (HighME = 120% requirement, *n* = 19) of predicted metabolizable energy requirements during late gestation for 52 d^1^

	Treatment			Parity			***P*-value** ^2^		
**Parameter** ^2^	LowME	HighME	SE	Heifers	Cows	SE	TRT	Parity	T × P
**Day** −**3 relative to calving**									
**Albumin, g/liter**	37.9	38.5	0.54	37.9	38.5	0.57	0.45	0.45	0.74
**Globulin, g/liter**	28.1	30.3	1.42	29.3	29.2	1.48	0.27	0.96	0.74
**Albumin: globulin ratio**	1.38	1.29	0.063	1.32	1.35	0.65	0.28	0.75	0.78
**Urea, m*M***	3.8	2.0	0.12	2.9	3.0	0.13	<0.01	0.55	0.07
**Glucose, mg/dL**	59.4	68.4	1.25	66.6	61.2	1.31	<0.01	0.02	0.09
**GGT, U/liter**	15.7	16.9	1.46	16.3	16.4	1.53	0.54	0.95	0.90
**AST, U/liter**	53.0	56.9	2.29	53.5	56.4	2.40	0.23	0.38	0.39
**GLDH** ^3^ **, U/liter**	9.6	9.7	(5.9–13.6)	13.0	7.7	(5.8–20.3)	0.95	0.08	0.50
**BHBA, µM**	263.8	281.8	13.10	242.8	302.8	13.70	0.33	0.01	0.16
**NEFA, µEq/liter**	474.4	288.0	37.13	331.0	431.4	38.83	<0.01	0.07	0.10
**Haptoglobin, g/liter**	0.14	0.13	0.010	0.14	0.14	0.010	0.57	0.92	0.66
**Cholesterol, mM**	2.0	2.3	0.10	2.2	2.1	0.10	0.04	0.68	0.31
**Insulin** ^3^ **, µg/liter**	2.5	2.0	(1.3–3.3)	1.93	2.55	(1.3–3.5)	0.43	0.29	0.68
**Day 7 relative to calving**									
**Albumin, g/liter**	36.9	39.0	0.60	37.8	38.1	0.68	0.03	0.81	0.57
**Globulin, g/liter**	31.1	33.6	1.39	32.0	32.7	1.46	0.20	0.69	0.28
**Albumin: globulin ratio**	1.20	1.18	0.051	1.20	1.19	0.054	0.73	0.92	0.16
**Urea, mM**	1.9	1.9	0.16	1.8	2.0	0.17	0.77	0.23	0.70
**Glucose, mg/dL**	61.2	68.4	1.57	66.6	63.0	1.64	0.01	0.08	0.92
**GGT, U/L**	16.8	18.9	1.51	17.1	18.6	1.58	0.34	0.47	0.37
**AST, U/liter**	69.4	71.4	2.71	65.8	75.0	2.83	0.59	0.03	0.55
**GLDH** ^3^ **, U/liter**	13.2	13.1	(8.6–17.5)	14.2	12.1	(8.6–19.2)	0.98	0.52	0.40
**BHBA, µM**	301.1	273.9	15.6	274.1	301.0	16.4	0.22	0.23	0.89
**NEFA, µEq/liter**	281.3	337.2	33.21	287.9	330.5	34.73	0.24	0.36	0.75
**Haptoglobin** ^3^ **, g/liter**	0.18	0.19	(0.14–0.24)	0.16	0.21	(0.13–0.26)	0.73	0.18	0.40
**Cholesterol, mM**	2.0	2.6	0.089	2.3	2.3	0.09	<0.01	0.68	0.66
**Insulin, µg/liter**	2.2	2.5	0.49	2.5	2.2	0.51	0.66	0.72	0.52

1 All cattle received the same ration after calving.

2 Treatment (TRT) and the treatment by parity interaction (T × P).

3 Data were normalized using an inverse (prepartum GLDH, prepartum insulin, and postpartum haptoglobin) or a logarithmic transformation (postpartum GLDH). The presented means are back-transformed. The 95% CI is shown in place of the SE.

At day 7 post-calving, cattle that had consumed HighME prior to calving had elevated (*P *≤ 0.03) serum albumin, glucose, and cholesterol concentrations than those fed LowME before calving. Serum GGT, AST, GLDH, NEFA, and haptoglobin concentrations and plasma insulin concentrations were similar (*P *≥ 0.20) between LowME and HighME cattle after calving. Cows had elevated (*P *= 0.03) serum AST by 9.2 ± 2.83 U/L compared to heifers, yet for heifers, serum glucose tended to be greater (*P *= 0.08) than that of cows. No differences were present (*P *≥ 0.18) between parities in the other blood analytes. The treatment by parity interaction did not affect (*P *≥ 0.16) postpartum blood analytes.

### Plasma glucose kinetics during the IVGTT

Cattle fed LowME and HighME differed (*P *= 0.02; Table 4) in their baseline glucose concentrations at the start of the IVGTT. However, glucose C_max_ and ΔC_max_ did not differ (*P *≥ 0.22) between treatments. Glucose clearance rate was similar (*P *= 0.21) for the two treatments and glucose positive I-AUC and total AUC were unaffected (*P *≥ 0.20) by prepartum ME consumption. When comparing heifers and cows, plasma glucose baseline concentration, C_max_, ΔC_max_, clearance rate, total glucose AUC, and positive glucose I-AUC were not different (*P *≥ 0.17). The treatment by parity interaction did not affect (*P *≥ 0.32) plasma glucose kinetics throughout the IVGTT.

**Table 4.** Plasma insulin, plasma glucose, and serum non-esterified fatty acid characteristics during an intravenous glucose tolerance implemented 7 d prior to calving with cattle that consumed rations formulated to provide a deficit (LowME = 80% requirements, *n* = 9) or an excess (HighME = 120% requirements, *n* = 8) of metabolizable energy relative to predicted requirements during late gestation for 52 d

	Treatment			Parity			*P*-value^1^		
Parameter	LowME	HighME	SE	Heifers	Cows	SE	TRT	Parity	T × P
**Plasma glucose, mg/dL**									
**Baseline**	55.9	61.0	1.34	59.5	57.3	1.40	0.02	0.25	0.89
**C_max_**	280.3	271.7	7.43	277.0	275.0	7.78	0.41	0.84	0.35
**ΔC_max_**	224.4	210.7	7.74	217.5	217.6	8.10	0.22	0.99	0.39
**Clearance rate, % × min** ^−^^**1**^	1.90	1.75	0.093	1.92	1.73	0.098	0.27	0.17	0.32
**Positive I-AUC, g/dL × min**	5.7	5.9	0.37	5.5	6.1	0.39	0.78	0.22	0.49
**Total AUC** ^2^ **, g/dL × min**	10.6	11.2	(10.1–11.9)	10.8	11.2	(10.1–11.8)	0.20	0.32	0.39
**Plasma insulin, µg/liter**									
**Baseline**	1.9	2.8	0.55	2.3	2.4	0.57	0.25	0.97	0.26
**C_max_**	9.9	10.4	1.48	10.2	10.0	1.55	0.79	0.92	0.61
**ΔC_max_**	7.9	7.6	1.26	7.9	7.7	1.3	0.85	0.89	0.91
**T_max_, min**	15.7	12.1	1.42	14.1	13.7	1.48	0.08	0.83	0.16
**Clearance rate, % × min** ^−^^**1**^	2.57	2.09	0.26	2.43	2.23	0.27	0.19	0.57	0.84
**Positive I-AUC** ^3^ **, µg/liter × min**	215.2	208.5	35.55	250.2	173.4	39.88	0.89	0.18	0.71
**Total AUC** ^3^ **, µg/liter × min**	366.1	431.3	50.18	349.0	448.4	56.29	0.36	0.22	0.60
**Serum NEFA, µEq/liter**									
**Baseline**	676.8	446.5	42.5	510.7	612.6	44.4	<0.01	0.10	0.66
**Max**	802.9	517.6	51.3	616.3	704.2	53.6	<0.01	0.23	0.64
**Min**	263.7	221.2	27.6	263.0	221.9	28.9	0.28	0.29	0.89
**GSRN, %**	58.4	43.9	5.44	42.4	59.9	5.68	0.07	0.03	0.47
**Disappearance rate, µEq/liter × min** ^−^^**1**^	5.0	2.5	0.53	2.8	4.7	0.57	<0.01	0.02	0.53
**dAUC, µEq/liter × min**	−23,150	−13,816	4,530.5	−16,895	−20,071	4,738.1	0.15	0.61	0.89
**Insulin sensitivity index** ^2^ ^**,**^ ^4^ **, min** ^−^ **^1^/(ng/liter)**	11.8	11.4	(1.6–22.1)	10.3	13.3	(2.1–24.5)	0.95	0.68	0.30

1 Treatment (Trt) and the treatment by parity interaction (T × P).

2 Data were normalized using an inverse transformation and presented means are back-transformed. The 95% CI is shown in place of the SE.

3 The model included day relative to calving as a covariate (positive insulin I-AUC: *P *= 0.012; total insulin AUC: *P *= 0.012).

4 Insulin sensitivity index was calculated according to Galvin et al. (1992) as modified by Pacini et al. (2009).

**Figure 3.**
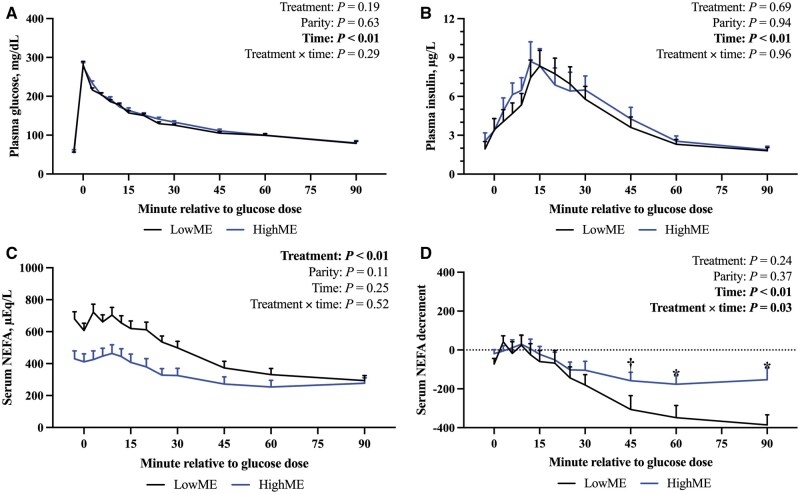
Plasma glucose (panel A), plasma insulin (panel B), serum non-esterified fatty acids (**NEFA**; panel C), and decremental serum NEFA (panel D) concentrations throughout an intravenous glucose tolerance test (**IVGTT**) performed 7 d prior to calving on cattle that were fed rations for 52 d prior to calving that were formulated to be deficient (**LowME **= 80% requirements) or in excess (**HighME **= 120% requirement) of predicted metabolizable energy requirements. The model also included the fixed effects of the treatment-parity interaction, parity-time interaction, and treatment-parity-time interaction. These factors were not significant (*P *≥ 0.17), and their *P*-values are not shown. Data are expressed as mean ± SE. Differences are determined when *P *< 0.05 (denoted using an asterisk) and tendencies are discussed when 0.05 ≤ *P *< 0.10 (denoted using a dagger).

Plasma glucose concentrations throughout the IVGTT for LowME and HighME cattle paralleled (treatment: *P *= 0.19; Figure 3A) each other. Glucose was drastically elevated (time: *P *< 0.01) at 0 min respective to the glucose bolus, and thereafter exponentially declined until 90 min, with no differences (treatment × time: *P *= 0.29) between treatments over time. The IVGTT plasma glucose concentrations were similar (parity: *P *= 0.63) for heifers and cows and did not differ (treatment ×parity: *P *= 0.47) between parities in a manner that was dependent on ME intake. Plasma glucose during the IVGTT was not affected by parity × time (*P *= 0.17) or treatment × parity × time (*P *= 0.91).

### Plasma insulin kinetics during the IVGTT

Cattle that were fed HighME rather than LowME tended to reach maximum insulin concentration earlier (*P *= 0.08) during the IVGTT. Otherwise, baseline insulin, insulin C_max_, insulin ΔC_max_, insulin clearance rate, positive insulin I-AUC, and total insulin AUC were not different (*P *≥ 0.19) between treatments. Neither parity nor the treatment × parity interaction affected (*P *≥ 0.16) IVGTT plasma insulin kinetics.

As with plasma glucose concentration during the IVGTT, plasma insulin concentrations changed (time: *P *< 0.01; Figure 3B) throughout the test, reaching maximal concentrations by 12 to 15 min after the glucose bolus and then gradually decreasing to a concentration similar to baseline at 90 min. Plasma insulin concentrations were not different (*P *≥ 0.53) between treatments, parities, or by the two- or three-way interactions of treatment, parity and time.

### Serum NEFA kinetics during the IVGTT

Feeding LowME prior to calving increased (*P *< 0.01) baseline and maximum serum NEFA concentrations and NEFA disappearance rate during the IVGTT relative to HighME. The GSRN tended to be greater (*P *= 0.07) for LowME compared to HighME cattle. There was no difference (*P *= 0.15) in the decremental AUC_0to90min_ between treatments. Heifers had reduced (*P *≤ 0.03) GSRN and NEFA disappearance rates respective to cows. Otherwise, baseline, maximum, and minimum serum NEFA concentrations did not differ (*P *≥ 0.11) by parity, nor (*P *= 0.61) did the decremental AUC_0to90min_. Serum NEFA characteristics during the IVGTT were comparable between parities of different treatments (treatment × parity interaction: *P *≥ 0.30).

Throughout the IVGTT, serum NEFA concentrations were substantially increased (treatment: *P *< 0.01; Figure 3C) when LowME instead of HighME was fed during late gestation. Non-esterified fatty acid concentrations did not change (*P *= 0.25) over time and were not different (*P *≥ 0.11) due to the effects of parity, treatment × parity, or treatment × time, parity × time, or treatment × parity × time. The decrement in IVGTT serum NEFA concentration diverged (treatment × time: *P *= 0.03; Figure 3D) at 60 and 90 min and between LowME and HighME cattle after the glucose dose, such that HighME cattle had a lesser (sliced by minute: *P *≤ 0.03) decrement in serum NEFA at 60- and 90-min. High ME cattle also tended to have a lesser (sliced by minute: *P *= 0.08) serum NEFA decrement 45 min after the glucose dose. Otherwise, the serum NEFA decrement varied (time: *P *< 0.01) throughout the IVGTT. Treatment, parity, and their two-way and three-way interactions with time had no influence (*P *≥ 0.24) on the IVGTT serum NEFA decrement.

## Discussion

We investigated how late gestation ME intake affects antepartum IR in heifers and cows respective to markers of inflammation and hepatic function. We had formulated rations to provide 80% and 120% predicted ME requirements prior to calving for LowME and HighME cattle, accounting for intentional body reserve mobilization and our retrospective analysis and nutritional modeling indicated that our ME targets were met. The HighME and LowME cattle consumed ME at 78% and 120%, respectively, of their predicted ME requirements and this was sufficient to produce differences in rump fat depth immediately prior to calving, adipocyte area, and metabolic indicators (NEFA and glucose) of energy deficit. Cattle that consumed LowME lost rump fat depth, whereas HighME cattle comparatively maintained rump fat depth and had greater adipocyte area before calving. No treatment differences were observed with respect to BW, demonstrating that LowME cattle compensated their deficient ME intake by mobilizing adipose reserves. In combination with LowME having elevated serum NEFA and lower serum glucose at 3 d before calving, the data indicate that our model sufficient induced a late gestation ME deficit for LowME cattle relative to HighME cattle.

An inherent consequence of LowME and HighME treatments is that the model-predicted MP supply was different (97% vs. 124% predicted MP requirements, respectively) due to the differences in DM and ME intake between treatments. While inevitable, this was not an intended experimental factor and, to counterbalance the difference in model-predicted MP supply, we formulated diets to be isonitrogenous for LowME and HighME treatments. Dietary CP was above current recommendations for beef cattle (i.e. 7% CP during mid-gestation, 9% CP, during late gestation, and 11% CP from calving to mid-gestation) and our prior work has shown that late gestation heifers had adequate MP supply when CP was 9.3% DM and ME was 96% predicted requirements (Hare et al., 2019b). It is therefore unlikely that the LowME cattle were truly deficient in MP supply. However, it is also doubtful that the HighME cattle had an advantage from consuming 13% more N than LowME cattle, since oversupplying MP did not improve BW, calf BW, or postpartum milk production in beef cattle and excess N was simply excreted (Hare et al., 2019a, 2019b). The proposition that model-predicted MP supply did not substantially bias treatment responses is supported by similar BW, conceptus-corrected BW, serum albumin and globulin, and calf birth BW between LowME and HighME cattle. Moreover, the magnitude of ME difference between LowME and HighME substantially exceeded the quantified difference in CP intake, supporting the conclusion that the differences in adiposity and metabolic indicators of energy balance were primarily driven by ME intake rather than protein supply.

Dairy cattle provided rations in excess of their requirements have reduced adipose-specific IR (Schoenberg and Overton, 2011; Schoenberg et al., 2012; Salin et al., 2017), although there is research to suggest otherwise (Mann et al., 2016b). The current study supports previous results that plasma glucose and insulin concentrations, and their maximum concentrations and clearance rates that define the AUC during an IVGTT, do not vary due to prepartum ME intake. As described in Hare et al. (2022), the conceptus sequesters large quantities of maternal glucose supply and may confound IVGTT glucose kinetics. That said, glucose clearance rate did not correlate to calf birth BW (ρ = 0.02, *P *= 0.89), and as such, plasma glucose concentration and AUC are likely to adequately reflect skeletal muscle IR. However, insulin insensitivity has been identified previously in dairy cattle by the NEFA decremental response during an IVGTT (Schoenberg and Overton, 2011; Schoenberg et al., 2012; Salin et al., 2017). A shallower NEFA decrement is interpreted to be adipose-specific insulin resistance. We found that greater ME intake resulted in a shallower NEFA decremental response, lower GSRN, and slower NEFA disappearance rate, in agreeance with prior work (Schoenberg and Overton, 2011; Schoenberg et al., 2012; Salin et al., 2017). These results suggest that feeding more ME diminished adipose-specific insulin responsiveness in late gestation beef cattle. It may be that the above-discussed unintentional discrepancies in MP intake could have influenced IVGTT responses within this study, as our previous work found that overfeeding MP to late gestation beef cattle increased baseline plasma glucose and insulin concentrations before an IVGTT (performed at 7 d prior to calving) and impacted indices of insulin resistance (Hare et al., 2022). However, in that study overfeeding MP did not influence glucose and insulin concentrations throughout the IVGTT, though, it should be noted that serum NEFA concentration and kinetics during the IVGTT were not measured. Importantly, cattle in that study were not losing condition prior to calving and had similar ME intake (Hare et al., 2019b), so comparison of IVGTT responses across studies should be interpreted cautiously.

A key component of our experimental design was to model overconditioned (BCS of 7, 9-point scale) late gestation beef cattle as losing body condition (2 BCS points over 100 d) prior to calving. The excess body condition reflected herd demographics at the Ontario Beef Research Center and formulating rations to accommodate BCS loss was intended to clarify how prepartum adiposity and the mobilization of adipose tissue relates to late gestation IR. Adiposity plays a central role in insulin sensitivity (Ghaffari et al., 2023) and over conditioning leads to elevated IVGTT glucose and insulin concentrations before calving in dairy cattle (Bogaert et al., 2018; Jaakson et al., 2018). If late gestation IR is altered in adipose tissue by ME consumption, then adipose reserves and mobilization (or deposition) must be accurately characterized. While treatments did not vary in body weight or rib and rump fat depth at the beginning of the study, the treatments caused LowME cattle to be leaner than HighME when the IVGTT was conducted. Therefore, in our experimental model, IVGTT responses should be impacted by differences in adiposity driven by dietary ME supply (Ghaffari et al., 2023) but, ultimately, these changes in adiposity were only reflected in IVGTT NEFA concentrations. By contrast, IVGTT glucose and insulin responses in this study were dissimilar to previous work demonstrating reduced IR and altered IVGTT glucose-insulin kinetics in overconditioned late gestation dairy cattle (Bogaert et al., 2018; Jaakson et al., 2018). This may be due to the timing of when IVGTT were performed relative to calving (1 wk compared to ∼3 wk before calving; Bogaert et al., 2018; Jaakson et al., 2018) or may be due to inherent differences in glucose-insulin kinetics between beef and dairy cattle. It could also be because the change in adiposity for cattle in the current study was purposefully caused through ME provision, as opposed to classifying cows by their condition without nutritional manipulation. Differences in the rate of mobilization of adipose tissue, as well as adipocyte size, have been linked to adipose-specific IR (De Koster et al., 2015, 2016). Maintaining or depositing adipose reserves prior to calving could plausibly diminish insulin signaling. Conversely, diet- and/or exercise-induced reductions in body reserves improves insulin sensitivity in other species, including humans, dogs, and rodents (Goodpaster et al., 1999; Bradley et al., 2008; German et al., 2009). With respect to ruminants, the is evidence that nutritionally induced BCS loss in mid- and late-pregnant sheep is associated with altered postprandial metabolite concentrations (Radunz et al., 2011). While these changes cannot be directly interpreted as altered insulin signaling, they do support the notion that changes in adiposity during late gestation could be linked to energy metabolism and, potentially, homeorhetic partitioning. Thus, the treatment-induced change in adipose mobilization in this study might give insight into the nature of prepartum adipose-specific insulin resistance.

While LowME cattle predominantly lost adiposity prior to calving, it is reasonable to consider whether skeletal muscle catabolism could have occurred provide amino acids (**AA**) for protein requirements or to augment energy supply. Transition dairy cattle are known to mobilize skeletal muscle during the close-up dry and fresh periods (Chibisa et al., 2008; Siachos et al., 2022) and oversupplying ME to late gestation dairy cattle allowed for an increase in longissimus dorsi diameter 10 d prior to calving (Mann et al., 2016a), suggesting that deficient ME intake could promote muscle protein mobilization in the current work. If AA were oxidized as ketogenic or gluconeogenic substrates, hepatic deamination could elevate plasma urea, as observed for LowME cattle. That said, the relative importance of AA as gluconeogenic substrates during the transition period is suggested to be minimal compared to alternative substrates, such as propionate and lactate (Aschenbach et al., 2010). Moreover, lower serum glucose and similar BHBA concentrations for LowME and HighME cattle do not support that skeletal muscle-derived AA partially compensated for glucose or ketone production. Although serum acetate and acetoacetate were not measured, and we cannot account for ketone utilization by tissues, plasma urea is not commonly independently used as an indicator of muscle catabolism because it is strongly influenced by diet (discussed below). Instead, body protein mobilization is typically characterized by performing ultrasonographic measurements of the longissimus dorsi (Mann et al., 2016a; Siachos et al., 2022), analyzing the expression of genes or abundance of proteins relating to muscle anabolism and catabolism (Chibisa et al., 2008; Mann et al. 2016a; Hare et al., 2019b) or markers of muscle protein degradation, such as creatinine and 3-methylhistidine (Jones et al., 1990; Phillips et al., 2003; Hare et al., 2019b), or measuring nitrogen balance (Chibisa et al., 2008; Hare et al., 2019b). None of these measurements were performed in the current study. Without them, we consider it premature to attribute the greater plasma urea for LowME cattle to skeletal muscle proteolysis, especially without corroborating differences in BW or serum albumin and globulin.

More plausibly, the greater plasma urea for LowME cattle reflect dietary formulation rather than muscle breakdown. Plasma urea N is positively associated with both dietary CP and ruminally degradable protein (**RDP**; Huntington et al., 2001; Bahrami-yekdangi et al., 2016; Hare et al., 2019b). However, few studies have isolated the independent effects of CP and RDP on urea metabolism in ruminants, and the reported responses are inconsistent. In both beef and dairy cattle, increasing dietary urea while maintaining CP has been shown to either increase (Thomas et al., 1984; Roseler et al., 1993; Broderick and Reynal, 2009) or not affect plasma urea concentration (Haig et al., 2002; Gleghorn et al., 2004; Vasconselos et al., 2009). These discrepancies may reflect differences in how studies managed associated changes in RDP and ruminally undegradable protein (Roseler et al., 1993; Haig et al., 2002; Broderick and Reynal, 2009) or, as acknowledged by Roseler et al. (1993), might relate to the ratio of CP relative to energy intake. The latter explanation is particularly relevant to our present work, where the increase in dietary urea N supply for LowME coincided with a substantial decrease in ME intake. The efficiency with which ruminally available N sources (soluble protein, amino acids, and non-protein nitrogen) are incorporated into microbial protein depends dietary energy intake (Bach et al., 2005). When readily fermentable carbohydrates are limiting, excess ruminally available N escapes assimilation into microbial protein, enters portal blood as ammonia, and is converted to urea in the liver (Reynolds et al., 1991). Thus, including urea in the LowME ration while simultaneously restricting ME likely created conditions that reduced the efficiency of microbial N utilization, thereby elevating plasma urea concentration.

Antepartum blood analytes did not support that feeding HighME caused inflammation or compromised hepatic function relative to LowME. Globulin and the albumin: globulin ratio are proposed as markers for inflammation (Bertoni et al., 2008; Trevisi and Minuti, 2018) and were not different between LowME and HighME cattle. In support, serum haptoglobin concentration was also similar for treatments, indicating that the difference in ME consumption did not influence systemic inflammation prior to calving. Examining other inflammatory markers not measured in the current study, such as the adipokines leptin, IL-1ß, plasminogen activator-inhibitor-1, and TNF-ɑ (de Castro et al., 2011; Depreester et al., 2018), would likely clarify whether local inflammation was present in adipose tissue as a consequence of prepartum ME consumption. Further, we did not observe differences between HighME and LowME cattle for serum GGT, AST, or GLDH (Bobe et al., 2004; Trevisi and Minuti, 2018) and this suggests that ME provision did not impair liver function. In fact, the greater serum cholesterol concentration for HighME cattle at 3 d prior to calving suggests that their hepatic function may have been enhanced relative to LowME cattle (Trevisi et al., 2009, 2012; Janovick et al., 2023). Though the greatest rates of cholesterol synthesis for ruminants occur in adipose tissue (Liepa et al., 1978), adipose tissue is considered a labile cholesterol reserve, while serum cholesterol reflects cholesterol homeostasis that is coordinated by changes in hepatic gene expression (Schlegel et al., 2012; Kessler et al., 2014). The role of the liver, as opposed to adipose tissue, in cholesterol homeostasis is further emphasized by LowME cattle having lower serum cholesterol despite mobilizing more rump fat tissue than HighME cattle. This response is consistent with previous research from dairy cattle (Drackley et al., 2014; Zenobi et al., 2018), as well as other research where feed restricted (80% of NRC nutrient requirements) close-up dairy cattle had markers of poorer liver function (Janovick et al., 2023).

We did observe moderate carry-over effects of prepartum nutrition 1 wk after calving. The greater serum glucose maintained for HighME cattle post-calving likely reflects that 7 d is not an adequate amount of time for LowME to adapt to the more fermentable postpartum rations. In addition to serum cholesterol remaining lower for LowME cattle, their serum albumin was also reduced. Albumin, as a negative acute phase protein, is considered a marker of impaired hepatic function as its decreased production indicates competition for substrates (Trevisi and Minuti, 2018). That said, LowME albumin concentration was not less than 35 g/liter (Trevisi and Minuti, 2018) and LowME cattle did not have other supporting markers, such as GGT, AST, and GLDH. If LowME feeding did affect liver function, it was not severe enough to impose a health risk or impair postpartum performance.

Beef cattle in the current study had similar DMI throughout the 52-d postpartum experimental period irrespective of prepartum ME intake. Apart from the decrease in rump fat depth with LowME feeding being maintained after calving, BW and rib fat depth were not different between treatments. In contrast, Boyd et al. (1987) found that supplementing late gestation beef cows with 2.7 kg/cow/d ground corn increased postpartum BW when compared with unsupplemented cows. Corah et al. (1975) reported that severe energy restriction (50% to 60% of requirements) reduced beef cow BW at weaning. However, Bohnert et al. (2013) observed in beef cattle that BCS at weaning tended to be increased with prepartum supplementation when cattle had greater BCS prior to calving, but that BW at weaning was not different due to supplementation regardless of prepartum BCS. Therefore, it may be that the excessive condition of cattle at the start of the experiment was sufficient to buffer the marginal ME deficit imposed on LowME cattle, such that they were capable of maintaining similar BW and rib fat depth relative to HighME cattle after calving. It is notable that neither LowME nor HighME lost condition after calving, as it demonstrates that beef cattle in the present work were not in a postpartum negative energy balance. Production responses might be different when cattle are properly- or under-conditioned.

### Parity responses

The difference in BW and fat thickness between heifers and cows is common and heifers were expected to be more insulin sensitive than cows due to their continued lean tissue accretion and lesser adiposity (Ghaffari et al., 2023). The differences in antepartum serum glucose and NEFA between cows and heifers align with what has been previously observed for dairy and beef cattle differing in parity (Wathes et al., 2007; Gärtner et al., 2019; Ferreira et al., 2021; Lean et al., 2023) and support that energy intake and metabolism differ for heifers due to their requirements for growth. These differences in requirements between heifers and cows might bias responses to prepartum ME intake. However, further work should be done to corroborate the treatment by parity tendencies that were observed for pre- and postpartum adiposity, as well as serum glucose and urea concentrations. While there were no overall differences between parities on glucose, insulin, or NEFA concentrations throughout the IVGTT, we uncovered lower GSRN and NEFA disappearance for heifers compared to cows. It is unclear why this response would occur, although it could suggest that heifers were less sensitive to insulin than cows. Alternatively, we speculate that the GSRN and NEFA disappearance rate may be proportional to the labile adipose reserves, since cows have greater adipose tissue mass. Additionally, serum AST was lesser postpartum for heifers than cows. The reason for this is unclear and we exercise caution in over-interpreting this response given that no other serum analytes were different between parities.

## Conclusion

Our findings indicate that late gestation ME supply was associated with modest differences in adipose tissue responsiveness to insulin, rather than systemic alterations in glucose or insulin kinetics. It is likely that the over conditioning of the cattle in this study paired with the intended mobilization of adipose reserves elicited this response. There was no indication that inflammation or impairment of liver function contributed to the altered IR between treatments. Although IR was affected, providing more ME prepartum did not impose a metabolic risk and enabled cattle to maintain improved body reserves after calving.

## Supplementary Material

skaf421_Supplementary_Data

## Abbreviations:

AA: amino acid
AST: aspartate transaminase
AUC_0to90min_: area-under-the-curve from 0 to 90 min
BHBA: beta-hydroxybutyrate
CCBW: conceptus-corrected BW
C_max_: maximum concentration
dAUC_0to90min_: decremental area-under-the-curve from 0 to 90 min
DMI: dry matter intake
GGT: gamma-glutamyl transferase
GLDH: glutamate dehydrogenase
GSRN: glucose-stimulated reduction in serum NEFA
HighME: high metabolizable energy
I-AUC_0to90min_: incremental area-under-the-curve from 0 to 90 min
IR: insulin responsiveness
IS_i_: insulin sensitivity index
IVGTT: intravenous glucose tolerance test
LowME: low metabolizable energy
ME: metabolizable energy
MP: metabolizable protein
NEFA: non-esterified fatty acids
RDP: ruminally degradable protein
T_max_: time to reach maximum insulin concentration
ΔC_max_: difference between C_max_ and baseline concentration
