# Supplementary material for: Late gestation metabolizable energy intake is associated with modest differences in adipose tissue insulin responsiveness in antepartum beef cattle
Source: J Anim Sci. 2026 Feb 16;104:skaf421. doi: 10.1093/jas/skaf421 (PMC12918310; doi:10.1093/jas/skaf421)
Supplement: skaf421_Supplementary_Data [file skaf421_supplementary_data.docx]

**SUPPLEMENTARY DATA**

**Late gestation metabolizable energy intake is associated with modest differences in adipose tissue insulin responsiveness in antepartum beef cattle^1^**

**Koryn S. Hare,*†^1^  Emily McKinlay,* Katharine M. Wood,* Gregory B. Penner,† and Michael A. Steele*^1^**

*Department of Animal Biosciences, Animal Science and Nutrition, Ontario Agricultural College University of Guelph, Guelph, ON, Canada N1G 1Y2, †Department of Animal and Poultry Science, College of Agriculture and Bioresources, University of Saskatchewan, Saskatoon, SK, Canada S7N 5A8

**^1^Corresponding authors:** [**masteele@uoguelph.ca**](mailto:masteele@uoguelph.ca), [**ksh249@usask.ca**](mailto:ksh249@usask.ca)

Journal of Animal Science

| **Table S1.** Model-predicted metabolizable energy (**ME**) requirements and supply of rations formulation to supply 80 (**LowME**, n = 19) or 120% (**HighME**, n = 19) of predicted ME requirements at d 270 relative to calving (d 275).^1^ | | | | | | | | | |
| --- | --- | --- | --- | --- | --- | --- | --- | --- | --- |
|  |  | ME Requirement, Mcal/d | |  | ME Supply, Mcal/d | |  | ME supply, %REQ | |
| Week relative to calving | Gestational day | LowME | HighME |  | LowME | HighME |  | LowME | HighME |
| -8 | 219 | 24.83 | 21.29 |  | 24.77 | 25.68 |  | 96.5 | 151.5 |
| -7 | 226 | 25.16 | 21.62 |  | 24.77 | 26.01 |  | 95.2 | 149.2 |
| -6 | 233 | 25.52 | 21.99 |  | 24.77 | 26.36 |  | 94.0 | 146.7 |
| -5 | 240 | 25.91 | 22.40 |  | 24.77 | 26.75 |  | 92.6 | 144.0 |
| -4 | 247 | 26.74 | 23.18 |  | 25.33 | 26.93 |  | 94.1 | 142.3 |
| -3 | 254 | 27.21 | 23.66 |  | 25.33 | 27.39 |  | 92.5 | 139.4 |
| -2 | 261 | 27.83 | 24.29 |  | 25.47 | 27.84 |  | 91.5 | 136.6 |
| -1 | 268 | 32.53 | 27.44 |  | 25.47 | 32.53 |  | 78.3 | 120.9 |
| 0 | 275 | 33.14 | 28.03 |  | 25.47 | 33.14 |  | 76.9 | 118.3 |
| ^1^Rations were formulated to facilitate the loss of 2 BCS points over 100 d and retrospectively analyzed with analysis from feed samples collected during the experiment and measured cow and calf birth BW. | | | | | | | | | |

| **Table S2.** Model-predicted metabolizable protein (**ME**) requirements and supply of rations formulation to supply 80 (**LowME**, n = 19) or 120% (**HighME**, n = 19) of predicted ME requirements at d 270 relative to calving (d 275).^1^ | | | | | | | | | |
| --- | --- | --- | --- | --- | --- | --- | --- | --- | --- |
|  |  | MP Requirement, g/d | |  | MP Supply, g/d | |  | MP supply, %REQ | |
| Week relative to calving | Gestational day | LowME | HighME |  | LowME | HighME |  | LowME | HighME |
| -8 | 219 | 613.5 | 589.4 |  | 690.0 | 892.6 |  | 112.5 | 151.4 |
| -7 | 226 | 626.8 | 602.8 |  | 689.3 | 891.9 |  | 110.0 | 148.0 |
| -6 | 233 | 641.7 | 617.8 |  | 688.5 | 891.2 |  | 107.3 | 144.3 |
| -5 | 240 | 658.3 | 634.5 |  | 687.7 | 890.4 |  | 104.5 | 140.3 |
| -4 | 247 | 667.9 | 661.2 |  | 706.7 | 913.8 |  | 105.8 | 138.2 |
| -3 | 254 | 688.5 | 681.9 |  | 705.7 | 912.8 |  | 102.5 | 133.9 |
| -2 | 261 | 707.5 | 707.5 |  | 709.6 | 917.9 |  | 100.3 | 129.7 |
| -1 | 268 | 732.9 | 732.9 |  | 708.4 | 916.8 |  | 96.7 | 125.1 |
| 0 | 275 | 760.8 | 760.8 |  | 707.2 | 915.7 |  | 93.0 | 120.4 |
| ^1^Rations were formulated to facilitate the loss of 2 BCS points over 100 d and retrospectively analyzed with analysis from feed samples collected during the experiment and measured cow and calf birth BW. | | | | | | | | | |





**Figure S1.** The treatment by day and parity by day interactions for prepartum BW (panels A and B) and prepartum conceptus-corrected BW (panels C and D). Cattle were provided rations formulated to supply either 80% (**LowME**, n = 19; white bars) or 120% (**HighME**, n = 19; blue bars) of predicted metabolizable energy requirements prior to calving. Interactions are sliced by the simple effect of day to provide mean separation for how BW and conceptus-corrected BW changed independent of each treatment and parity throughout the prepartum period. Data were normalized using a logarithmic transformation. The presented means are back-transformed, and error bars represent the 95% confidence interval. ^a-d^Means that do not share a common lowercase superscript differ (*P* < 0.05) for HighME cattle. ^ABC^Means that do not share a common uppercase superscript differ (*P* < 0.05) for LowME cattle.


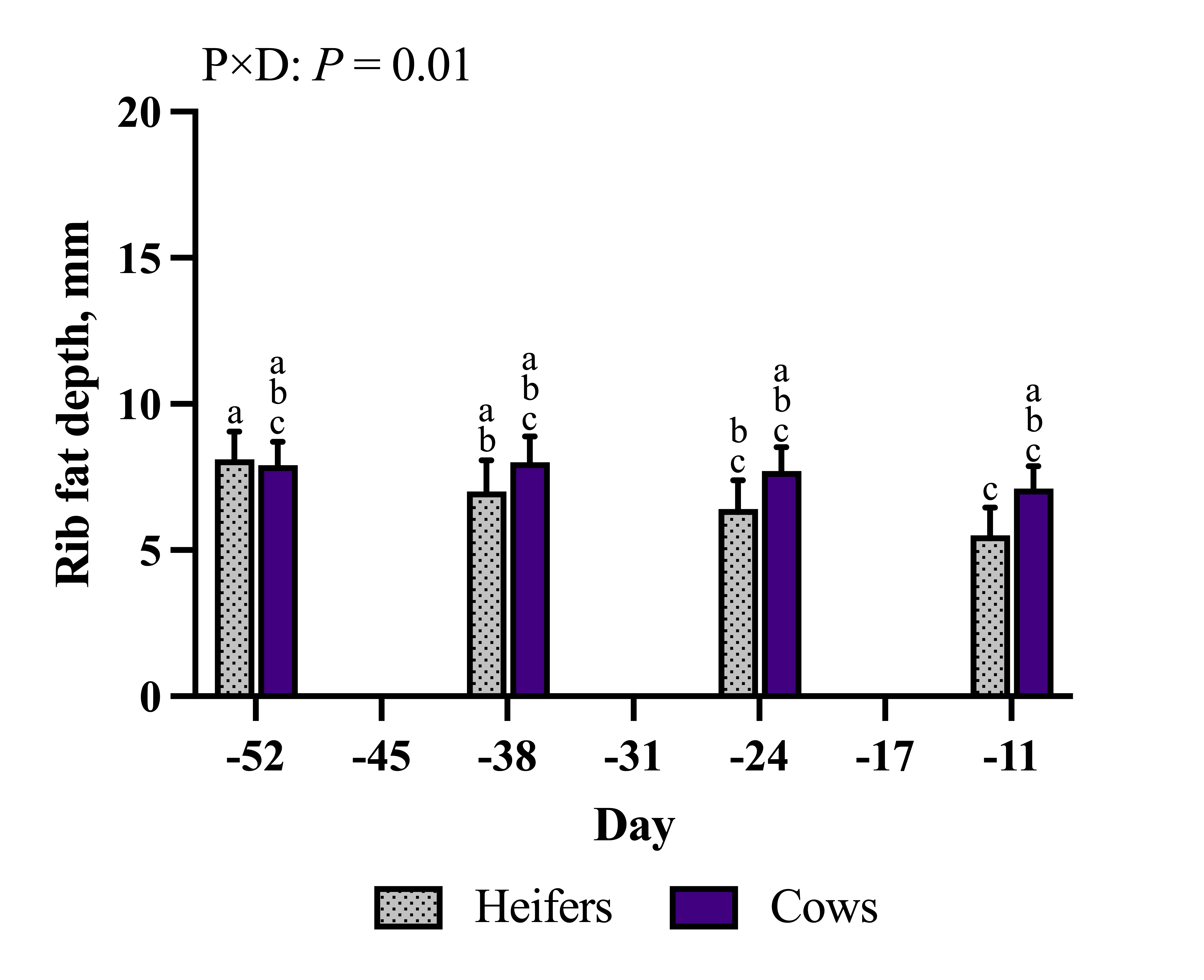


**Figure S2.** The interaction of parity by day (**P×D**) for prepartum rib fat depth (*P* = 0.01) of heifers (black-dotted gray bars) and cows (purple bars) that were fed rations formulated to supply either 80 or 120% of predicted metabolizable energy requirements for 52 d prior to calving. Data are presented as mean ± SE. ^abc^Means that do not share a common lowercase superscript differ (*P* < 0.05).
